# Electric field down-regulates CD9 to promote keratinocytes migration through AMPK pathway

**DOI:** 10.7150/ijms.42840

**Published:** 2020-03-15

**Authors:** Ran Ji, Miao Teng, Ze Zhang, Wenping Wang, Qiong Zhang, Yanling Lv, Jiaping Zhang, Xupin Jiang

**Affiliations:** 1 Department of Burn and Plastic Surgery, The First Affiliated Hospital of Chongqing Medical University, Chongqing, 400016, China.; 2 Institute of Burn Research, State Key Laboratory of Trauma, Burns and Combined Injury, Southwest Hospital, Army Medical University (Third Military Medical University), Chongqing 400038, China.; 3 Department of Plastic Surgery, State Key Laboratory of Trauma, Burn and Combined Injury, Southwest Hospital, Army Medical University (Third Military Medical University), Chongqing 400038, China.

**Keywords:** CD9, electric field, AMPK, cell migration, wound healing

## Abstract

Endogenous electric field (EF)-directed keratinocytes migration is known to play a key role in the wound re-epithelialization process. Although many molecules and signaling pathways are reported important for directional keratinocytes migration under EF, the underlying mechanism remains unclear. Our previous research found that CD9, a trans-membrane protein, is involved in wound re-epithelialization and CD9 downregulation contributes to keratinocytes migration. In this study, we observed the effect of EF on CD9 expression and keratinocytes migration. The keratinocytes migrated directionally toward the cathode and CD9 expression was down-regulated under EF (200mV/mm). In addition, CD9 overexpression reversed EF-induced migratory speed and the electrotactic response of keratinocytes. Also, we found that EF reduced AMP-activated protein kinase (AMPK) activity. Furthermore, AICAR, an AMPK activator, increased CD9 expression under EF, while compound C, an AMPK inhibitor, decreased CD9 expression in keratinocytes. Our results demonstrate that EF regulates CD9 expression and keratinocytes directional migration, in which AMPK pathway plays an important role.

## Introduction

Skin is the largest organ of human body with numerous functions. Endogenous EF is widespread in biosphere and is produced instantaneously after the skin disruption which was found at the middle age of 19th century as Emil Du-Bois Reymond, a German Physiologist, detected a weak current with a self-made galvanometer 1.The wound edge acts as the anode of the endogenous EF until wound re-epithelialization is completed 2, 3. For rat corneal wound, the currents take 60-90 minutes to reach a peak, and then decreased until the wound is healed 4. As for skin wound, endogenous EF intensity maintained at about 150-200mv/mm for three days after injury, and then decreased gradually, disappeared completely following wound closure 5. EF has a great influence on wound healing 6. Some studies have shown that when EF was removed, the wound healing speed was 25% slower 7. Clinical trials using EF to stimulate wound healing have reported a significant increase in the healing rate 8. The migration of keratinocytes toward the wound core site is a critical step of wound repair. EF is considered to be the most important directional factor to guide directional migration of keratinocytes 9. Additionally, PI3K and PTEN signaling are involved in the process of electrotactic cell migration. PI3K accumulates in the front of migrated cell and PTEN is distributed in the back as a negative regulatory protein of PI3K 3. Also, inhibiting the activity of EGFR kinase alone reduces directional movement indicated that EGFR redistribution is associated with the directional migration 10. Therefore, endogenous EF is an important event to wound healing but how it regulates the migration of keratinocytes remains unsolved.

CD9, a 24 kDa cell surface protein, is a member of integral trans-membrane proteins 11, was firstly found expressed in hematopoietic system cells, and in various types of cells participating in a variety of cellular biological processes, including regulating sperm-egg fusion, tumor migration, cell mobility, adhesion and differentiation 12. Our previous study showed that wound repair was delayed in CD9-null mice and the expression of CD9 in keratinocytes was down-regulated after injury and then increased, reaching the same level as normal when the wound was healed 13. All the results indicated that CD9-downregulation is required for re-epithelialization. There is a certain time-space correspond between CD9 and EF strength change, thus, we hypothesize that CD9 is involved in EF-guided keratinocytes migration during wound healing.

AMPK, an AMP-dependent protein kinase, is a key molecule in the regulation of bioenergy metabolism. AMPK can be activated by various stimuli, including cell pressure, exercise, many hormones and substances affecting cell metabolism 14-16. In addition to its role in metabolism, AMPK is closely related to cell migration and plays diverse roles in different cells and environments 17. Studies have shown that the activation of AMPK inhibits the migration of tongue squamous cell carcinoma cells 18. Loss of AMPK activation promotes the invasion and metabolism in many cancers 15, 19. Recently, an AMPK activator met has been shown to reduce cell proliferation and delay wound healing 20, suggesting that AMPK may be involved in the cytobiology of keratinocytes during wound repair. Coincidentally, AMPK participates in epidermal migration in the early stage of wound under hypoxic environment, and AMPK inhibition significantly enhances the motor ability of keratinocytes 21. Additionally, AMPK activity in tibialis anterior (TA) muscle was attenuated under high-frequency electric stimulation 22. All these studies have raised an indication that AMPK pathway may be related to EF-promoted keratinocytes migration.

In the present study, we used mouse keratinocytes (MKs) and HaCaT cells to observe the expression of CD9 change under EF simulating for different time *in vitro*. Our results revealed that EF down-regulated the expression of CD9 in keratinocytes, promoted cell motility and lateral migration and inactivated AMPK pathway, while AMPK activation reversed the effect of EF on CD9 expression. In conclusion, our results suggested that EF down-regulated CD9 in keratinocytes through AMPK pathway. Our findings explain the importance of CD9 in EF-induced keratinocytes directional migration and provide new insights into the mechanism of EF-guided cell migration during wound healing.

## Materials and methods

### Ethics statement

All animal-based investigations were designed and performed in accordance with the Guide for the Care and Use of Laboratory Animals published by the National Institutes of Health (NIH Pub. No. 85-23, revised 1996). The entire project was reviewed and approved by the Animal Experiment Ethics Committee of the Third Military Medical University in Chongqing, China.

### Cell culture and chemical treatments

HaCaT cells were obtained from Cell Bank of the Chinese Academy of Sciences in Beijing, China. Primary mouse keratinocyte (MKs) were cultured using a described method before 23. Briefly, neonatal Balb/c mice (postnatal day 1-3) mice were immersed in 75% alcohol for 2-3 minutes, and then rinsed with PBS for 2-3 times. Gradually blunt skin separation with scissors and tweezers. After repeated washed by PBS, adding neutral protease II (1x) until submerged the skin, 4°C for the night. After the epidermal and dermis were separated, the epidermis was cut in sterile PBS, digested with 0.25% trypsin/ 0.02% EDTA solution for 2-3 minutes, then neutrallized with 1640 medium containing 10% fetal bovine serum and 1% penicillin/streptomycin, and filtered with a sieve after blowing, centrifugated for 5min. MKs can be inoculated after resuspending with 1640 medium. Both HaCaT and primary mouse keratinocytes were cultured in 1640 medium (SH30809.01B, Hyclone, USA) containing 10% fetal bovine serum (S-FBS-500, Scitecher, USA) and 1% penicillin/ streptomycin (GA3502, Genview, Australia), digested with 0.25% trypsin/0.02% EDTA solution (SH30042.01, Hyclone, America). Cell cultures were performed in a 5% CO2 atmosphere at 37°C. Cells were treated with 1mM AICAR (ab120358, Abcam, UK), 2μM Compound C (ab120843, Abcam, UK).

### EF stimulation and imaging of single-cell motility

The EF strength (200mV/mm) was based on previously study 24. EF stimulation was given as previously described 25. Briefly, keratinocytes were stimulated by EF through two silver electrodes immersed in Steinberg's solution (60 mM NaCl, 0.7 mM KCl, 0.8 mM MgSO4, 0.3 mM CaNO3·4H2O, and 1.4 mM Tris base, pH 7.4) which was connected to the culture medium by two agar bridges. During the stimulation of EF, the time-lapse imaging was performed using a Zeiss imaging system (Carl Zeiss Meditec, Jena, Germany), and the images were acquired every 5 minutes to observe the motility of single cells visually. Images were analyzed by Image J.

### Recombinant adenovirus vector for CD9 overexpression

Ad-CD9-GFP and CD9 mock vector Ad-GFP were purchased from Shanghai GeneChem, Co. Ltd. (Shanghai, China). Briefly, HaCaT cells inoculated into 6-wells plates, infected by Ad-CD9-GFP and CD9 mock vector Ad-GFP for 48h. Observe the transfection under fluorescence microscope and proved by Western blot. The molecular weight of CD9-GFP is about 55KD.

### Quantitative analysis of cell migration

We quantified the electrotaxis and motility of cells with Image J software. We track the location of the nucleus every 5 minutes and locate the starting point at the origin. The directional expression of cell migration is cosθ, where θ is the angle between the EF direction and the straight line from the beginning to the end of the cell. Cosθ is calculated by the displacement of cell migration. Near 0 represents the random cell movement, near 1 represents the cell moving towards the cathode, and near -1 represents the cell moving towards the anode. Cosθ ranges from -1 to +1, objectively quantifying the direction of cell migration. The X-axis velocity is the X value of the end position divided by time, representing the migration velocity along the EF vector. Direction multiplied by displacement velocity reflects the displacement velocity of the element. The displacement velocity is the linear distance between the start and end positions of the element divided by time. The trajectory velocity is the total length of the trajectory divided by time, reflecting the movement of cells.

### Western blots analysis

Extracted protein was resolved in SDS-PAGE Gel, transferred to polyvinylidene difluoride membrane. After blocked by 5% non-fat milk, adding diluted primary antibody, 4℃ for the night, and incubated with corresponding secondary antibodies at room temperature for 1h. The Molecular Imager ChemiDoc TMXRS+ Imaging System (Bio-Rad) and chemiluminescene reagents were cooperated to detect the signal. The using of primary antibodies was as follows: CD9 (1:1000, 13174S, Cell Signaling Technology, USA), AMPK (1:1000, ab32047, Abcam, UK), p-AMPK (1:1000, ab23875, Abcam, UK), GAPDH (1:5000, HRP-60004, Proteintech, USA).

### Statistical analysis

Data are represented as mean ± standard error of mean (SEM). Two-tailed Student's t-tests and one-way ANOVA were performed to determine the significant differences using the SPSS statistical software (Chicago, IL, USA). P < 0.05 is considered statistical significance.

## Results

### Keratinocytes were promoted to migrate from positive to negative under EF stimulation

**Figure 1:**
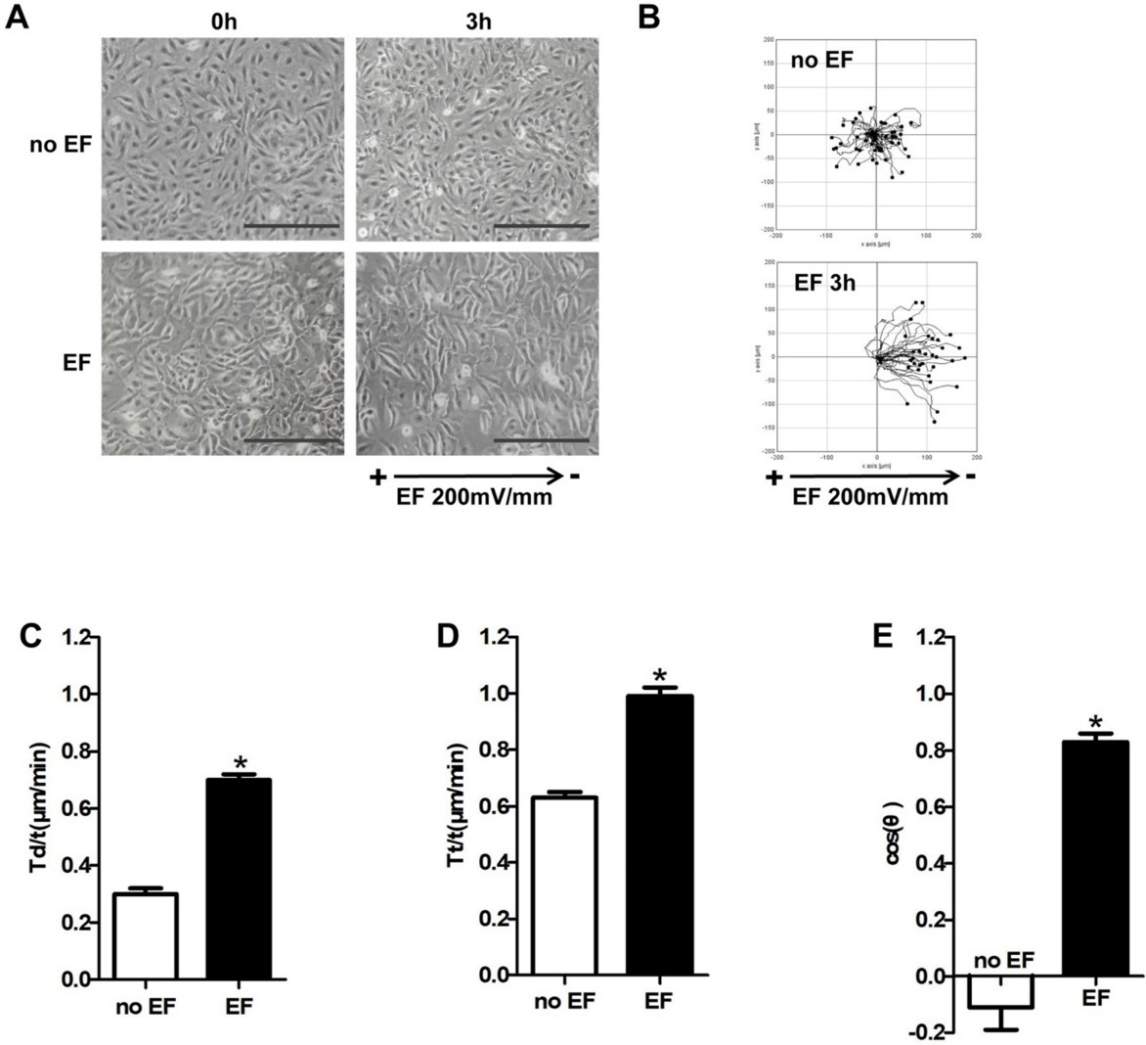
**The effect of EF stimulation on keratinocytes migration**. **(A)** HaCaT cells were stimulated with (3h) and without current EF. Pictures were taken both before and after the EF treatment. Scale bar: 100μm. **(B)** The trajectories of cells for 3h recorded by time-lapse microscopy. **(C-E)** Quantitative analysis of Tt/t (µm/min), Td/t (µm/min) and directness (cosθ) of keratinocytes migration. The data was shown as the mean±SEM (n=3). *, p<0.05 compared with no EF group.

In order to examine whether EF stimulation could cause keratinocytes directional migration, we used time-lapse microscopy to observe the trajectory of HaCaT cells under EF. As shown in Figure 1A, HaCaT cells showed a significant change in arrangement after EF stimulation (200mV/mm), from irregular to longitudinal, which indicated that the device can effectively electrified cells (Figure 1A, 1B, Movie 1-1). HaCaT cells in normal state moved closely to their starting position with a small range without EF. However, after 3 hours of EF treatment, the motion range of has increased significantly, and cells showed a clear tendency to move towards negative pole (Figure 1B, Movie S1-2). Statistical analysis showed that displacement velocity Td/t (μm/min) and trajectory velocity increased after EF treatment (Figure 1 C, D). EF stimulation made cosθ close to +1 (Figure 1E). So EF stimulation promoted keratinocytes to move straightly toward cathode, and increased the cell migration velocity.

### CD9 was down-regulated in keratinocytes under EF stimulation

**Figure 2:**
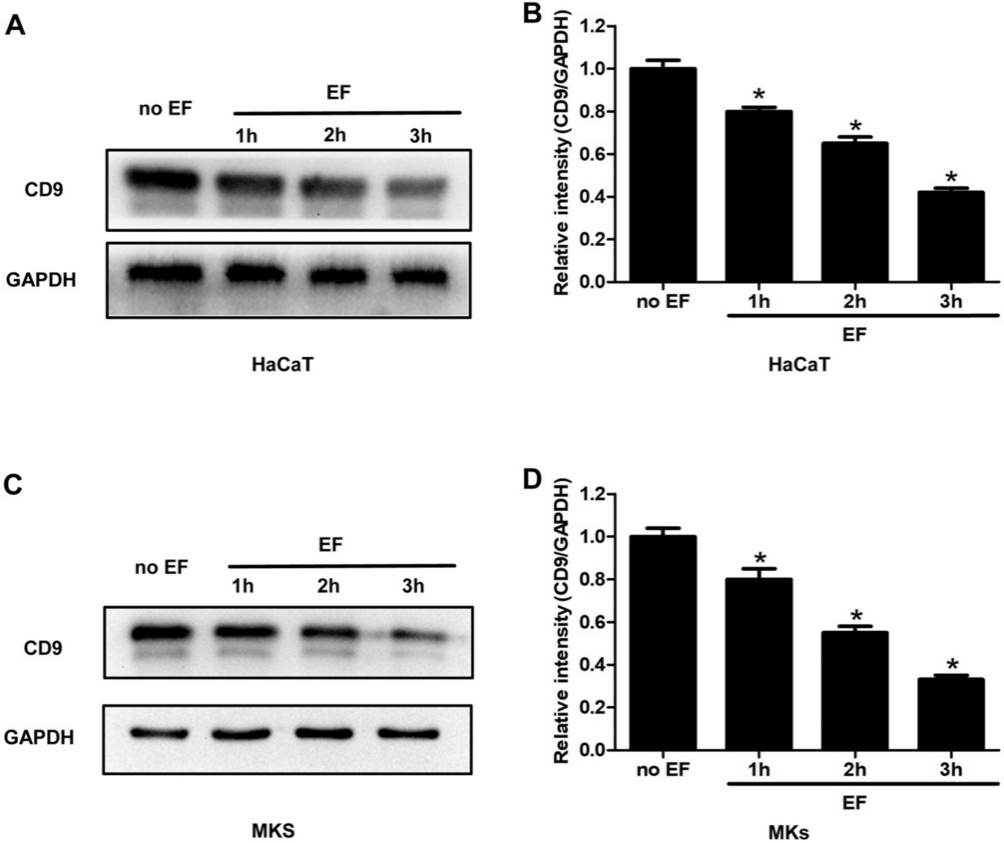
** The effect of EF on CD9 expression in keratinocytes**. **(A, C)** The expression of CD9 in HaCaT cells and MKs were determined by western blot. **(B, D)** The results were quantified by relative intensity. The data was shown as the mean±SEM (n=3). *, p<0.05 compared with no EF group.

As an exogenous current signal stimulus, EF processing is bound to transmit this signal through some molecules in the cells, causing its directional migration. To examine whether CD9 was involved in EF-induced migration, HaCaT cells and MKs cells were electrified for 1h, 2h and 3h. The expression of CD9 protein was analyzed using western blot. It was found that CD9 showed a high expression in untreated cells, but a lower level after EF treatment (Figure 2A, C). Quantitative analysis showed that after 1h, 2h, 3h of EF treatment CD9 protein level in HaCaT cells decreased by 20%, 35%, and 58%, and in MKs cells by 20%, 45%, and 67%. (Figure 2B, D). The results indicated that EF down-regulated CD9 expression and it is time-dependent.

### CD9 inversely regulated EF-guided keratinocytes migration

**Figure 3:**
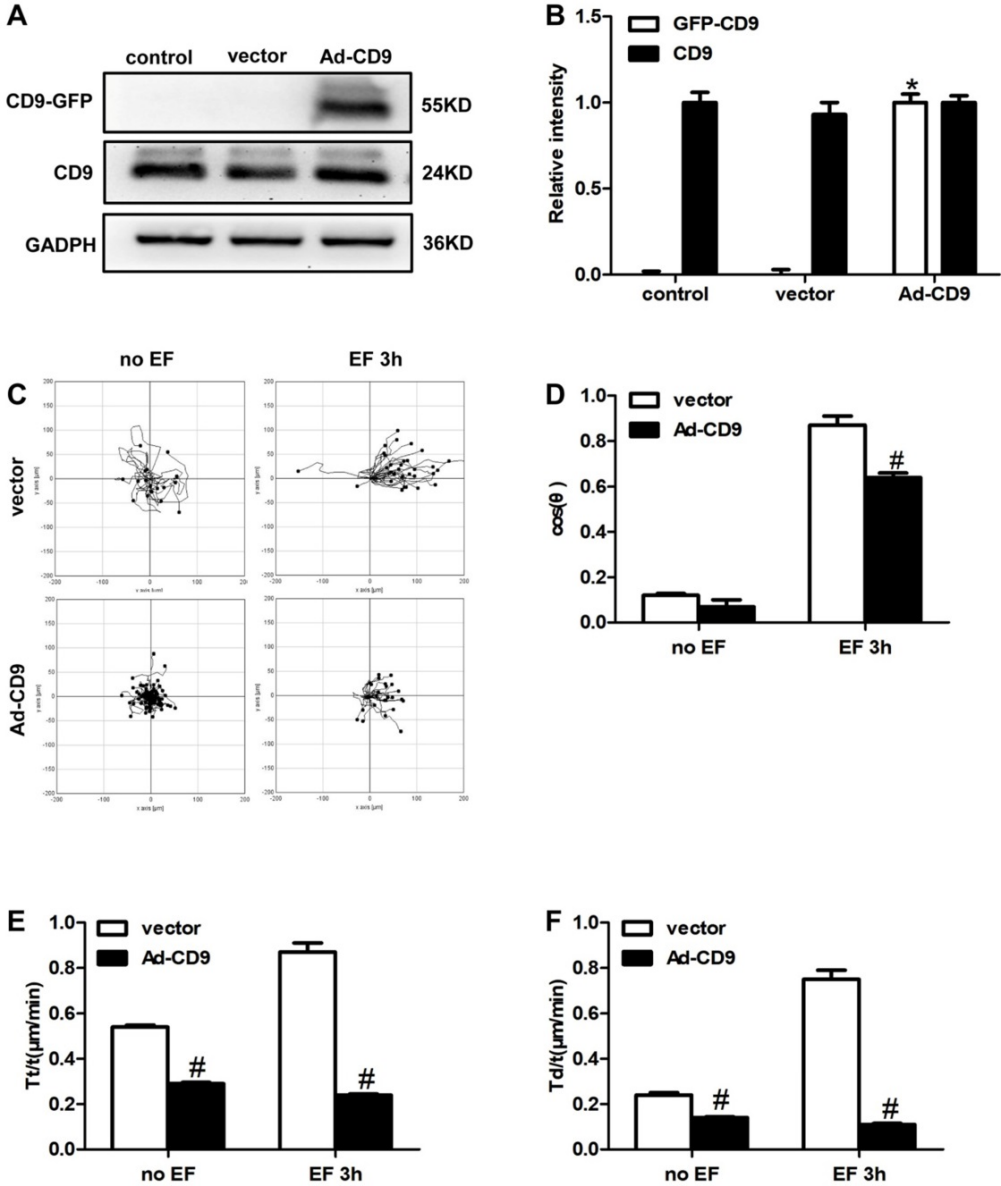
** The effect of CD9 on EF-promoted keratinocytes directional migration. (A, B)** The expression of CD9 and CD9-GFP were determined by western blot. The data was shown as the mean±SEM (n=3). *, p<0.05 compared with vector and control group. **(C)** Cell migration was recorded by time-lapse microscopy at 1 frame every 5 minutes and was analyzed by Image J. **(D-F)** Quantitative analysis of cosθ, Tt/t (μm/min) and Td/t (μm/min) of keratinocytes migration. The data was shown as the mean±SEM (n=3). #, *p*<0.05 compared with vector group.

The above results showed that EF treatment down-regulated the expression of CD9. Reverse intervention to regulate protein expression is a classic and traditional method to study the effect of target proteins; therefore, we used adenovirus (Ad) as a vector to increase the expression of CD9. To examine whether CD9 was involved in EF-guided keratinocytes migration, recombinant adenovirus vectors for overexpressing CD9 (Ad-CD9) were constructed and used to infect HaCaT cells prior to EF treatment. The effects of adenovirus infection were quantified and verified before further experiments. After infection for 48 hours, more than 90% of the keratinocytes confirmed to be infected by observing GFP expression using a fluorescent microscope (Supplementary Figure S1). The effective overexpression of CD9-GFP fusion proteins was confirmed using western blot (Figure 3A, B). As shown in Figure 3C, CD9 overexpression decreased the cells moving range. Statistic results showed that CD9 overexpression decreased the EF-induced keratinocytes directedness (Cosθ) by 26% (Figure 3D, Movie S2-2 and Movie S3-2), reduced trajectory velocity (Tt/t) and the displacement velocity (Td/t) under EF by 72% and 85%, while only 46% and 41% under no EF (Figure 3E, 3F, and Movie S2-1 and Movie S3-1). We also proved that adenovirus transfection had no effect on both keratinocytes velocities and cosθ, compared with control group (Supplementary Figure S2). Besides, the cell viability have no difference among the control, vector and Ad-CD9 group (Supplementary Figure S3). All the results showed that CD9 overexpression reduced the migration velocity of keratinocytes in EF, and also affected electrotaxis to some extent.

### The activity of AMPK in keratinocytes was inhibited by EF stimulation

**Figure 4:**
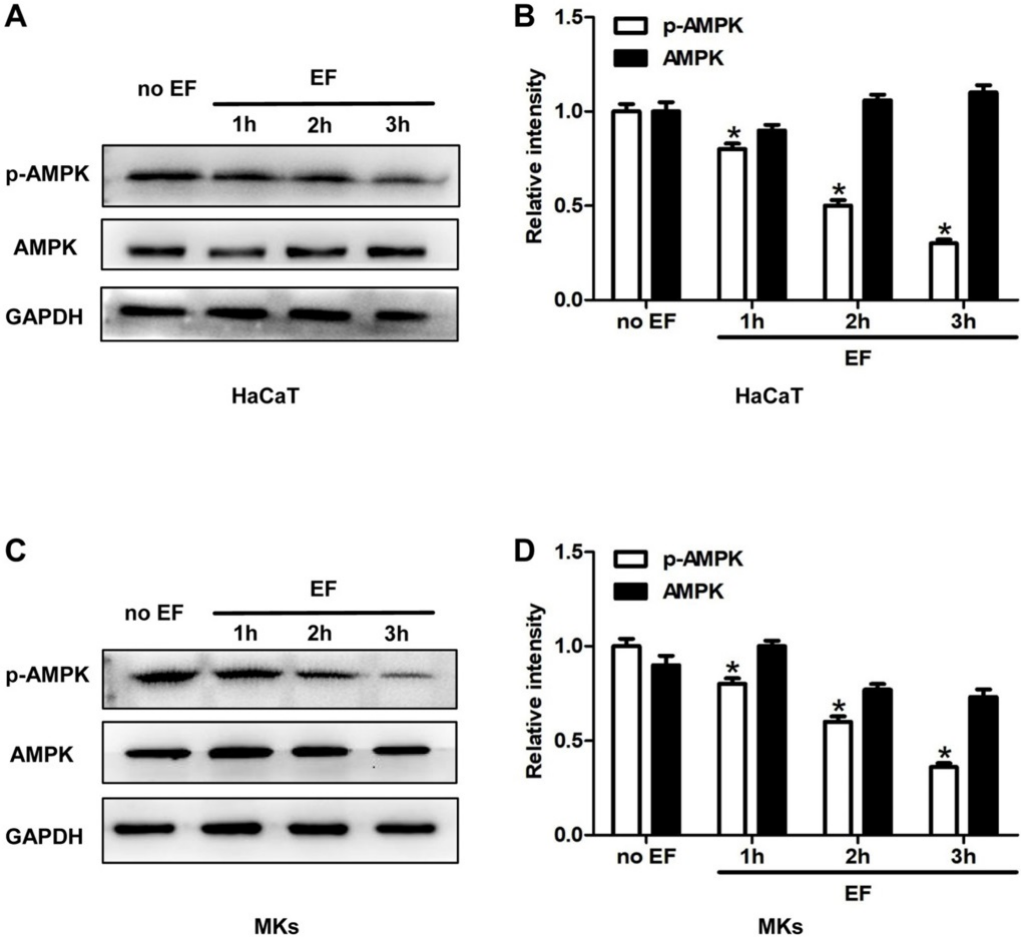
** The AMPK signaling was inhibited by EF in keratinocytes. (A, C)** The levels of p-AMPK and AMPK in HaCaT cells and MKs were tested by western blot. **(B, D)** The results were quantified by relative intensity. The data was shown as the mean±SEM (n=3). *,* p*<0.05 compared with no EF group.

To investigate whether a correlation existed between AMPK pathway and the CD9 expression and cell migration, we tested the status of AMPK signaling in HaCaT cells and MKs under EF for 1h, 2h, 3h. Phosphorylated AMPK (p-AMPK) and AMPK were detected using immunoblotting. The phosphorylation of AMPK (p-AMPK) in keratinocytes showed a high level in untreated cells and was decreased gradually after EF treatment in both HaCaT cells and MKs cells (Figure 4A, 4C). Quantitative analysis showed that under EF treatment for 1h, 2h and 3h, the p-AMPK level in HaCaT cells decreased by 18%, 53%, and 70%, and in MKs cells by 22%, 40%, and 64%, while AMPK level had no siginicant difference in neither HaCaT or MKs cells (Figure 4B, 4D). It is suggested that the activity of AMPK was inhibited by EF stimulation and was time-dependent.

### AMPK signaling was involved in EF-induced CD9 expression changes in keratinocytes

**Figure 5:**
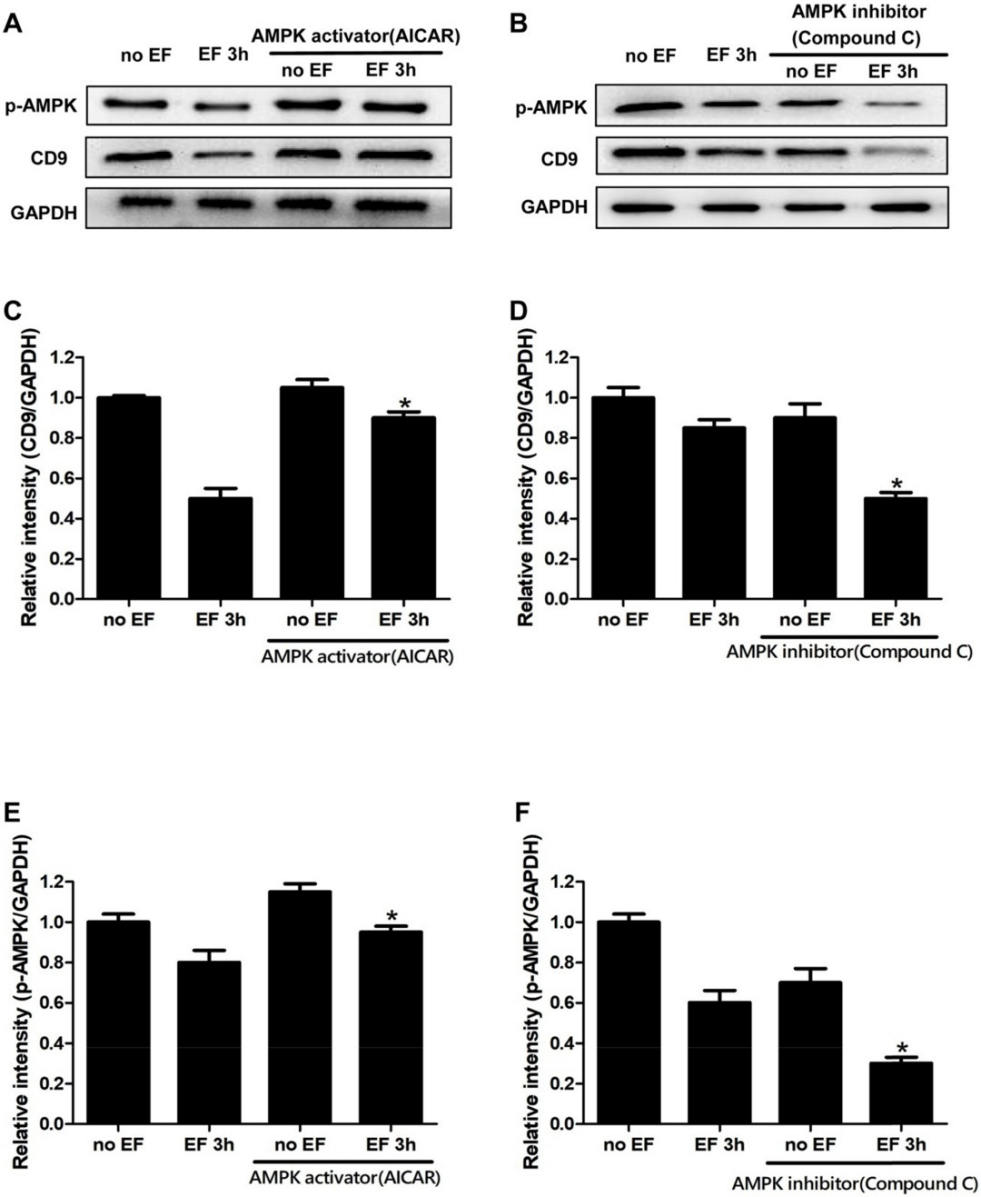
** Involvement of the AMPK pathway in EF-regualated CD9 expression in keratinocytes. (A, B)** The levels of p-AMPK and CD9 in HaCaT cells were determined by western blot. **(C, D, E, F)** The results intensity was quantified by relative intensity. The data was shown as the mean±SEM (n=3). *,* p*<0.05 compared with EF 3h group.

Through the above experiments, we found that the expression of CD9 decreased significantly after EF treatment. In order to study whether the change of CD9 depends on AMPK pathway, we then tested the expression of CD9 in EF-treated keratinocytes after activating AMPK signaling with AICAR or inhibiting AMPK signaling with Compound C. The effective activation or inhibition of AMPK pathway was confirmed using western blot (Figure 5A, 5B and 5E, 5F). The CD9 protein level of keratinocytes in EF 3h+AICAR group showed 1.8-fold increase in relative to EF 3h group, while the CD9 protein level of keratinocytes in no EF+AICAR group only showed 1.05-fold increase in relative to no EF group (Figure 5C). We then further inhibited the AMPK pathway using Compound C, which resulted in a reduction in the CD9 protein levels by 70% in EF-treated keratinocytes, while it was reduced by 30% under no EF conditions (Figure 5D). Additionally, the levels of p-AMPK and AMPK in CD9-overexpressed HaCaT cells were also detected and the result showed that CD9 overexpression did not active or inactive AMPK signaling in HaCaT cells (Supplementary Figure S4). All these results suggested that the AMPK pathway plays a crucial role in EF-regulated CD9 expression.

## Discussion

For wound healing, accumulating evidence proves that EF is the most significant factor to keratinocytes migration. In order to repair wounds effectively, endogenous EF, which formed immediately after skin disruption, guides keratinocytes to migrate directionally. However, the mechanism of how EF guides keratinocyte migration remains unknown. We previously demonstrated that the downregulation of CD9 was critical to initiate keratinocytes migration during wound healing 14, 26. In the current study, we confirmed that EF downregulated CD9 expression and promoted keratinocytes directional migration via AMPK pathway.

The process of keratinocytes migrating into the wound center is an essential step of skin wound healing. EF is generated when a wound occurs that disrupts the epithelial barrier 26. During wound healing, the current flows can be tightly regulated in space and time. Spatially, the strongest currents are found at the wound edge, while lower magnitude currents flow in the wound center. Temporally, electric currents at wounds appear immediately after wounding, slowly rise to the peak and then keep decreasing gradually until disappear completely when the wound closed 2, 26. Recently, it has been reported that the electrotaxis is mediated by multiple signaling pathways that include membrane protein, like EGFR and integrins, PI3 kinases/PTEN, cAMP, Rho small GTPases 2.

Tetraspanins family, as trans-membrane proteins, mainly associate with other tetraspanins, integrins and signaling receptors, thereby forming tetraspanin-enriched microdomains on the cell surface 27. Tetraspanin CD9 is a key molecule that contributes to the transformation of cell migration phenotype and promotes cell migration 13, 28, 29. Here, we found that EF downregulated CD9 expression in keratinocytes and CD9 overexpression reversed EF-induced migratory speed and the electrotactic response of keratinocytes. Our previous study revealed that CD9 was downregulated following wounding, which promoted keratinocyte migration during wound healing 23. Besides, CD9 silencing triggered the switch from integrin αvβ5 to αvβ6 in keratinocytes 30. These results indicated that CD9, a kind of membrane protein, acts as a crucial role in EF-guided keratinocytes migration. Meanwhile, our results also partially showed the mechanism of how CD9 expression is regulated during wound healing.

Interestingly, we found that EF inactivated AMPK signaling, which was related to CD9 expression in keratinocytes. It has been indicated that AMPK pathway is involved in cell migration in many types of cancer cells 19, 31, and previous study has confirmed that the inhibition of AMPK promotes the mobility of HaCaT cells 17. Furthermore, hypoxic preconditioning also promotes the migration of keratinocytes through AMPK pathway 32, 33. In this study, we confirmed that endogenous EF reduced the CD9 expression in keratinocytes and inhibited AMPK pathway. Thus, we pharmacologically activated AMPK with AICAR and inhibited AMPK with compound C, to observe the expression changes of CD9 levels in keratinocyte under EF. We found that the activation of AMPK signaling increased CD9 expression in keratinocytes under EF, while AMPK inhibition decreased it. These findings indicated that the AMPK pathway was also involved in the process of EF-regulated CD9 expression in keratinocytes. Indeed, after the skin defection, the wound microenvironment is complex, including hypoxia, endogenous EF and so on 2, 32. Combining with the results of our study, both hypoxia and endogenous EF promote keratinocytes migration through AMPK pathway, which also emphasized the importance of AMPK signaling. Further work is required to elucidate the possible effect of CD9 in keratinocytes migration under hypoxic and EF microenvironment. As we known, slow-healing wound cause by any kinds of factors is very likely due to the decrease of migration speed or ability of keratinocytes 34. As for clinical application, EF has been indicated useful for chronic wound to close the wound in several clinical trials 8, 35. The healing rate of ischemic skin ulcers treated with electric current is about twice that of ulcers untreated with EF 36. Since our study revealed the role of CD9 in EF-guided keratinocyte migration and its possible mechanism, it may enrich the molecular mechanisms of EF promoting chronic wound healing and be able to provide a new clinical treatment idea.

In conclusion, this study demonstrates that EF down-regulated CD9 expression by inhibiting AMPK pathway activation, which promoted keratinocytes migratory speed and the electrotactic response. These results provided new insights into the molecular mechanism of EF-guided keratinocytes directional migration during wound healing.

## Supplementary Material

Supplementary figures and movie legends.

Supplementary movie 1-1.

Supplementary movie 1-2.

Supplementary movie 2-1.

Supplementary movie 2-2.

Supplementary movie 3-1.

Supplementary movie 3-2.

## Abbreviations

EF: electric field
AMPK: AMP-activated protein kinase
GAPDH: glyceraldehyde-3- phosphate dehydrogenase
GFP: Green fluorescent protein
Tt: trajectory distance
Tt/t: trajectory velocity
Td: displacement distance
Td/t: displacement velocity
MKs: mouse keratinocytes
