# Supplementary material for: Electric field down-regulates CD9 to promote keratinocytes migration through AMPK pathway
Source: Int J Med Sci. 2020 Mar 15;17(7):865–73. doi: 10.7150/ijms.42840 (PMC7163358; doi:10.7150/ijms.42840)
Supplement: Supplementary file 1 — Supplementary figures and movie legends. [file ijmsv17p0865s1.pdf]

Supplementary Material

Supplementary figures

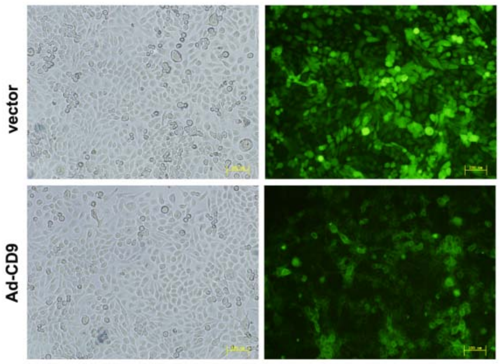

Figure S1. **HaCaT cells were infected with recombinant adenovirus vectors for overexpressing CD9 (Ad-CD9) and negative vectors (vector).** HaCaT cells were infected with mock vector (vector) or Ad-CD9 for 48 h and then observed under fluorescence microscope to determine the infection efficiency by visualizing expression of the gene for GFP. Bar=200μm.

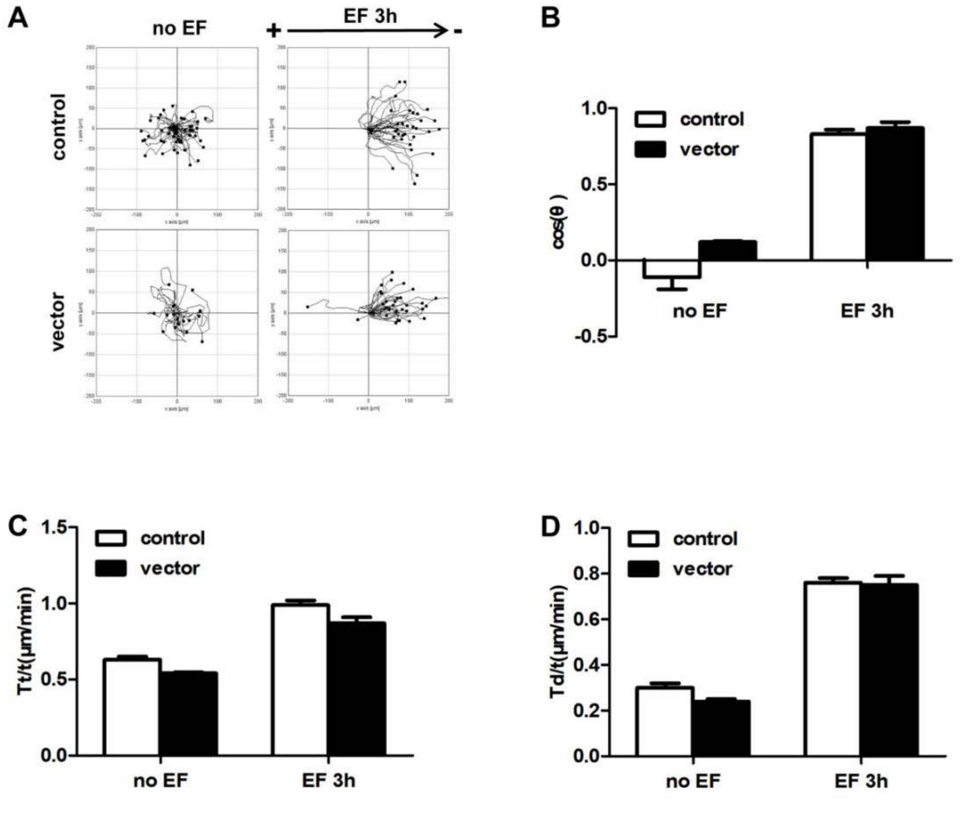

Figure S2. The effect of vector transfection on keratinocyte motility. **(A)** Trajectories of control and vector group. **(B)** Cosθ of HaCaT cells were shown as mean ± SEM, showed no statistical difference between control and vector group. **(C)** Trajectory velocity (Tt/t) of HaCaT cells were shown as mean ± SEM, showed no statistical difference between control and vector group. **(D)** Displacement velocity (Td/t) of HaCaT cells were shown as mean ± SEM, showed no statistical difference between control and vector group.

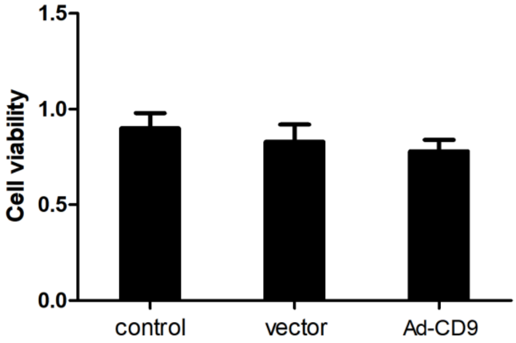

Figure S3. Cell viability assay. Normal HaCaT cells (control) and HaCaT cells infected with recombinant adenovirus vectors for CD9 overexpression (Ad-CD9) and negative vectors (vector) for 48 hours. Cell viability was determined by using Cell Counting Kit-8 (CCK-8; Dojindo Molecular Technologies, Rockville, MD, USA) according to manufacturer's instructions. The experiment was repeated three times. Graph represents the X±SEM (n = 3). No difference in cell viability was detected in these groups.

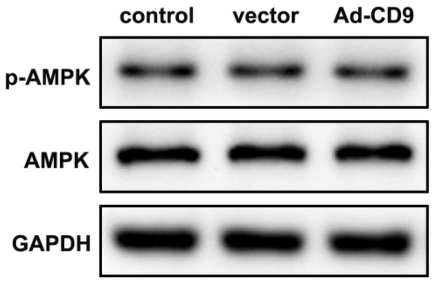

Figure S4. The effect of CD9 on AMPK signaling in HaCaT cells. Normal HaCaT cells (control) and HaCaT cells infected with recombinant adenovirus vectors for CD9 overexpression (Ad-CD9) and negative vectors (vector) for 48 hours. The levels of p-AMPK and AMPK in HaCaT cells were tested by western blot.

Supplementary movies

Movie S1-1. HaCaT cells were plated onto slide and visualized by time-lapse microscopy for 3 hours.

Movie S1-2. HaCaT cells were plated onto slide, treated with EF (200mV/mm) and visualized by time- lapse microscopy under EF for 3 hours.

Movie S2-1. HaCaT cells transfected with recombinant adenovirus mock vectors (Vector) were plated onto slide and visualized by time-lapse microscopy for 3 hours.

Movie S2-2. HaCaT cells transfected with recombinant adenovirus mock vectors (Vector) were plated onto slide, treated with EF (200mV/mm) and visualized by time-lapse microscopy under EF for 3 hours.

Movie S3-1. HaCaT cells transfected with recombinant adenovirus vectors for overexpressing CD9 (Ad-CD9) were plated onto slide and visualized by time-lapse microscopy for 3 hours.

Movie S3-2. HaCaT cells transfected with recombinant adenovirus vectors for overexpressing CD9 (Ad-CD9) were plated onto slide, treated with EF (200mV/mm) and visualized by time-lapse microscopy under EF for 3 hours.
